# Efficiency of Illusory Choice Used as a Variant of Situation Selection for Regulating Emotions: Reduction of Positive Experience But Preservation of Physiological Downregulation

**DOI:** 10.1007/s10484-020-09484-x

**Published:** 2020-08-08

**Authors:** Simon Thuillard, Elise S. Dan-Glauser

**Affiliations:** Institute of Psychology, University of Lausanne, Lausanne, Switzerland

**Keywords:** Emotion regulation strategies, Situation selection, Illusory choice, Emotional responses, Physiological arousal

## Abstract

*Situation selection* is an emotion regulation strategy consisting in choosing a future emotional situation. Past research showed that *Situation selection* triggers a decrease in negative experience, exocrine reactions and respiratory activity, while maintaining stable positive experience. In this study, we wanted to replicate these observations and test emotional responses that follow an *Illusory choice*, i.e., when the chosen situation is not available and replaced by another. Sixty-eight participants watched emotional pictures, either in a condition in which the images were imposed, or in a condition in which they could perform a choice. In these latter trials, participants saw either the chosen option (*Situation selection*) or the non-selected option (*Illusory choice).* Continuous recordings of experience and physiological arousal showed that, unlike *Situation selection*, *Illusory choice* decreased positive experience but not negative experience. Strikingly, however, we showed that having the choice decreased skin conductance and respiratory arousal, regardless of whether the choice was respected or not. These results have important implications regarding emotion regulation through *Situation selection*, since having the choice about the upcoming emotional situation, no matter if we really end up in this situation, gives a sense of control that may be sufficient to alleviate physiological responses to stressors.

## Introduction

Throughout our life, personal decisions shape our living style, social networks, professional situations, and relationships. The ability to make those decisions strengthens our sense of control. Since Seligman argued (Seligman 1975) that control is fundamental to avoid learned helplessness, many studies have examined the impact of this sense of control on human behaviour. In particular, sense of control has been shown to promote mental and physical health, career success, and functional cognitive processing (Christensen et al. 1991; Thompson and Spacapan 1991; Declerck et al. 2006). However, not all decisions are alike. One particular choice we can make as human beings is to decide what emotional situations we will experience in the near future. This kind of choice has been identified as a potentially efficient emotion regulation strategy, called *Situation selection*. *Situation selection* “involves taking actions that make it more (or less) likely that we will end up in a situation we expect will give rise to desirable (or undesirable) emotions.” (Gross and Thompson 2007; see also Jazaieri et al. 2017). It has been experimentally demonstrated that *Situation selection* can be an effective emotion regulation strategy, particularly on physiological changes such as skin conductance, and respiration reactivity (Thuillard and Dan-Glauser 2017), which are important parameters for the understanding of emotion in general and emotion experience in particular (Christopoulos et al. 2019; Hameed et al. 2019). Transposed to real-life situations, one could therefore assume that having the choice regarding an upcoming emotional situation will have positive emotional consequences. However, due to the organizational and social constraints of today’s lifestyle, it is not always possible to obtain what we choose. This is what we have called “*Illusory choice*”, a counterpart of *Situation selection,* where a choice is made but the situation we end up in is not the expected one. The present article reports on a study that examined the differential consequences of *Situation selection* and *Illusory choice* on the unfolding of the emotional process. Additionally, this study seeks to determine whether the functionality of *Situation selection* relies on choosing potentially advantageous situations, or whether the sense of control given by the action of choosing, no matter if we end up living what we have chosen, is already regulatory.

Emotion is a central aspect of life (Bradley et al. 1996, 1993). It generally follows an assessment of a situational trigger (*appraisal* processes), which in turn generates changes in three major emotional response systems: experience, expressivity, and physiological arousal (Levenson 1994; Lang 1995; Gross 2014). Emotion rarely occurs without individuals attempting to modify its unfolding and outcomes (Tomkins 1984). This *emotion regulation* can be achieved by acting on the situation, on the attention or the meaning we attribute to it, or on the emotional responses that are triggered (Gross 1998). Effective emotion regulation plays a crucial role in healthy adaptation (Gross and John 2003; Gross and Muñoz 1995) and social functioning (Eisenberg et al. 2000); whereas difficulties in emotion regulation have been associated with pathologies such as anxiety and mood disorders (Campbell-Sills and Barlow 2007). It is therefore crucial to identify how and to which extent each emotion regulation strategy is effective.

The Process Model of Emotion Regulation (Gross 2001, 2007) is one of the earliest and most influential emotion regulation models in the field. It models five emotion regulation strategies dispatched along the emotion generative pathways, indicating the time of action of each in the process. When studying the efficiency of these different strategies to regulate emotion, research has most often focused on contrasting two of them (*Reappraisal* and *Suppression*). Results of these studies suggest that *Reappraisal* is more adaptive than *Suppression* (Webb et al. 2012), potentially because the earlier a strategy intervenes in the emotion generative process, which is the case of *Reappraisal* as compared to *Suppression*, the more likely it is to be effective (Sheppes and Gross 2012). Following this rationale, we assume that the first strategy to be represented in the model, namely *Situation selection,* should be particularly effective in regulating emotion. By choosing whether to live a situation or not, individuals modify their future emotional unfolding, thus achieving emotion regulation.

*Situation selection* was first considered as a unitary concept. However, it has recently been suggested there may be two mechanisms by which *Situation selection* could be an effective strategy (Thuillard and Dan-Glauser 2017). The first one is related to the chosen situation. After comparing their different options, people generally choose the most beneficial one and eventually experience a more positive situation. The second mechanism by which we assume *Situation selection* is effective is through the empowering action of making a decision about our own emotions. Below, we briefly review the main findings regarding these two potential mechanisms and propose a follow-up to further investigate the second one.

There is already evidence for the first mechanism in the literature. Sands and Isaacowitz (2016) showed that the choices in a *Situation selection* procedure are guided by the information that can be obtained on two dimensions of affects (Russell 1980; Bradley and Lang 1994): the arousal and valence levels of these situations. The authors concluded that people generally engage in positive rather than negative situations and, if faced with several positive situations, will choose the less arousing one. The chosen (more positive) situation thus intrinsically leads to less negative states (Livingstone and Isaacowitz 2015). In another study where patterns of choice were investigated, results showed that what people choose and the time spent interacting with the given material depend on the interaction of age and control beliefs (Rovenpor et al. 2012). Overall, these results show that the situation that is selected heavily depends on the intrinsic features of the options and the choosing individuals.

It could also be argued that controlling the upcoming emotional situation is already regulatory per se. Hence, regardless of the options presented or selected, emotional responses to two identical situations could be different if we have chosen the situation or not. To our knowledge, only one study has examined this question to date. In this study, a special within-subject protocol was used to contrast reactions to *identical* emotional stimuli in two different conditions: (a) when a stimulation is chosen and then presented, and (b) when the same stimulation is presented without any prior choice procedure (Thuillard and Dan-Glauser 2017). Results showed that having the choice in negative situations decreased negative experience by 6%. Additionally, and although the choice task increased skin conductance at the beginning of the negative emotional stimulation, it took only 7 s for the skin conductance level to reach a lower level than in the non-choice condition. Similarly, respiratory rate showed a significant decrease in the choice condition, while it remained stable in the non-choice condition. Interestingly, choice procedure also enhanced expressivity. In contrast, choosing a positive image before seeing it left the experience unaffected and generally reinforced its calming effect regarding physiological reactions. Once again, the design in that past study permitted to compare the effect of identical emotional stimuli in the “Chosen” and “Imposed” conditions, enabling the authors to highlight the pure effect of choosing, and not the effect of a different resulting situation.

Considering the use of *Situation selection* in real-life situations, it could happen that even if we opt for attractive situations (or less deleterious), we may end up not receiving what we choose. We call this *Illusory choice*. Concepts related to *Illusory choice* have already been used in the field of economics in relation to decision making and, closer to psychology, while testing sustained attention or motivational processes. For example, it has been shown that choosing a task increases the effort an individual is willing to invest (Dember et al. 1992), regardless of the task that is ultimately performed. Even more relevant, another study has shown that inducing the illusion of control (by letting people choose a lottery ticket) modifies beliefs about future gains (Langer 1975). Therefore, it seems likely that *Illusory choice* may also impact the way emotional situations are experienced. However, to our knowledge, *Illusory choice* as such has never been operationalized in research on emotional processes.

The present research was designed with two objectives. First, we wanted to replicate the effect of *Situation selection* on emotion responses with another sample. Second, we wanted to further test the effect of choice by investigating *Illusory choice,* in which we can choose an upcoming emotional situation (as when performing *Situation selection*), but in which the choice is ultimately not respected. In such cases, does choice retain its regulatory effect? Or, on the other hand, does non-respected choice provoke counteracting effects and reinforce the negative emotions into play? To test this question, we used a *within-subject design* and induced emotions by presenting emotional pictures, as already used in numerous studies (Dan-Glauser and Gross 2011; Dan-Glauser and Scherer 2011; Bradley et al. 1993; Codispoti et al. 2001). We tested a condition of *Situation selection,* in which people could perform a choice about the emotional content of the upcoming image. In this case, the chosen option is ultimately presented. We additionally included a condition called *Illusory choice,* in which participants performed the same type of choice but were confronted to the non-chosen image. Both types of choice were compared to imposed situations (no choice of upcoming situation). There are two possible outcomes: (1) the impact of the *Illusory choice* condition on emotional responses remains similar to that of the *Situation selection* condition. In this case, we could assume that choice alone is already regulatory, regardless of the situation it leads to; or (2) the two conditions give different outcomes, for example by inducing increased arousal for people who did not obtain what they asked for. This would mean that proposing *Situation selection* as a way to regulate emotions would have a beneficial effect only if we are able to offer the chosen option. Given the positive impact of *Illusory choice* on cognitive behaviours and beliefs (see previous paragraph), we expect to find the first option, i.e., there should be no significant interaction between choice (given or not) and respect of this choice (respected or not), replicating the previously found effects (Thuillard and Dan-Glauser 2017). Namely, we expect:

- For negative images: a decrease in negative experience and in exocrine and respiratory activity, and an increase in negative expressivity for all the chosen conditions.
- For positive images: an enhanced cardiovascular reaction and exocrine activity, coupled with a decrease in respiratory rate for all the chosen conditions.

The timing of emotional response development is important in describing emotional arousal unfolding (Stein et al. 1993; Esslen et al. 2004). Moreover, specific dynamics regarding emotional reactions to pictures have been previously identified (Bradley and Lang 2000; Codispoti et al. 2001). It is therefore crucial to consider response dynamics when investigating fast emerging emotion responses to pictures. This is particularly true when studying the impact of emotion regulation, which has shown differential dynamics depending on the considered responses. (Dan-Glauser and Gross 2011; Thuillard and Dan-Glauser 2017). We therefore also included a time factor in our design to identify and isolate effects that are only transient, or that appear only after a few seconds of viewing. Our intent was not to investigate when specific differences occur, but rather to identify and interpret any difference occurring during the presentation window.

## Methods

### Participants

A power analysis, with a power of 0.8 (Cohen 1988), effect sizes derived from partial eta squares of previous similar study (f = 0.20), and α = 0.05, yielded a target sample size of 63. To compensate for potential technical difficulties or signal artifacts, a sample size of 70 participants was targeted. Participants had to participate to two separate sessions, a questionnaire session and a testing session. Seventy-one participants were invited to the first session. Three of them were excluded because they did not return for the second session. Hence, 68 participants participated fully to our study (34 males and 34 females). Participants were all first-year Psychology students participating for course credits. Participants were recruited during a psychology course with a brief description of the project, without mentioning that emotion regulation efficiency was the target of the study. Participants ranged in ages between 18.0 and 26.8 years, with an average of 21.1 years (SD = 1.7 years). Exclusion criteria were pregnancy, medication, and a current diagnosis of anxiety or mood disorder. Inclusion criteria were ages between 18 and 45 years old and general good health. With respect to this latter criteria, participants were tested with the 12-Item Short-Form Health Survey (SF-12, Ware et al. 1996) and scored an average of 77.5% (SD = 9.1) of good health (100% being excellent health at the mental, physical, and social levels). Since handedness may have an influence on emotion processing and physiological outputs (Reuter-Lorenz et al. 1983; Bourne 2008), and shows differences regarding difficulties in emotion regulation (Mohammadi et al. 2015), all participants had to be right-handed. Their scores on the Edinburgh Handedness Inventory (Oldfield 1971), scoring -100 for totally left-handed and + 100 for totally right-handed respondents, averaged 71.8 (SD = 19.1).

### Operationalization of *Situation Selection* and *Illusory Choice*

In the present study, we used a similar within-subject paradigm as in Thuillard and Dan-Glauser (2017). *Situation selection* was operationalized by asking participants to choose between two picture categories (see the *Stimuli* section). Participants pressed a key to choose a picture category and a corresponding picture was almost immediately presented. In addition to the *Situation selection* condition, we implemented an *Illusory choice* condition, in which a choice was given, but the picture that was *not* chosen was subsequently presented. These two *Choice* conditions were contrasted to a condition in which participants were not given the opportunity to choose (*Imposed* condition).

**Fig. 1:**
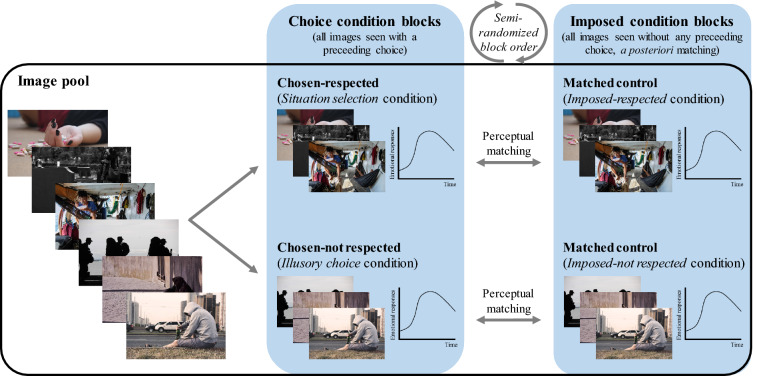
Illustration of the trial separation to test the *Situation selection* and the *Illusory choice* effects. From the image pool, some pictures were seen in the *Situation selection* condition (middle section, Chosen-respected section), and some pictures were seen in the *Illusory choice* condition (middle section, Chosen-not respected section). All images were also seen under an *Imposed* condition, where participants were presented with a picture without performing any prior choice. *A posteriori*, imposed trials were divided into two categories: (a) imposed pictures that were also seen in a *Situation selection* condition (matched control images named *Imposed-respected* condition, upper right section) or (b) imposed pictures that were also seen in an *Illusory choice* condition (matched control images named *Imposed-not respected* condition, lower right section). Please note that images shown here are for illustration purpose and are not part of the stimuli used. We show here illustration of the trial separation for negative images. Same procedure is applied for positive images. Each participant had a different distribution of images in each of the *Choice* conditions

Each stimulus was shown twice to each participant: once in the *Choice* conditions (either in the *Situation selectio*n or in the *Illusory choice* condition), and once in the *Imposed* condition. To assess the respective impact of *Situation selection* and of *Illusory choice*, the recorded trials in each of these two categories were compared to a matching set of trials recorded under the *Imposed* condition, as illustrated in Fig. 1. To characterize the effect of *Situation selection*, trials recorded under the *Situation selection* condition (chosen-respected trials) were compared to trials with the same stimuli (i.e., with the same visual characteristics) recorded under the *Imposed* condition (forming the so-called *Imposed-respected* image control group, Fig. 1 top right). To characterise the effect of *Illusory choice*, trials recorded under the *Illusory choice* condition (chosen-not respected trials) were compared to trials with the same stimuli recorded under the *Imposed* condition (forming the so-called *Imposed-not respected* image control group, Fig. 1 bottom right). Image examples in Fig. 1 illustrates this: when we contrasted imposed-respected trials and *Situation selection* trials (Fig. 1, upper section), we actually compared the reactions to the same pictures. This means that any difference could only be attributed to the effect of choice. This is also true when contrasting imposed-not respected trials and *Illusory choice* trials (Fig. 1, lower section).

### Stimuli

Eighty seven images were taken from the Geneva Affective Picture Database (GAPED, Dan-Glauser and Scherer 2011), which gathers negative and positive stimuli, categorized as such based on mean valence and arousal ratings of the database. Stimuli could further be included in different labelled categories. These labels are necessary to allow participants to perform a choice based on written descriptors of the upcoming situations. Negative pictures consisted of four content types: spiders, snakes, animal mistreatment and human mistreatment. These words were used as labels to offer a choice between categories in the *Choice* conditions. Positive pictures also consisted of four categories: landscapes, human babies, mammals (generally cute offspring), and sport (inspirational) pictures. Since examples of the latter content type are rare in the GAPED, we added nine pictures of sport pictures from the International Affective Picture System (IAPS, Lang et al. 1999). Labels used in the choice procedure for positive categories were “Landscape”, “Baby”, “Mammal”, and “Sport”. Thus, of the final 96-picture pool, 48 were negative and 48 were positive, with 12 pictures of each content type. We presented emotional stimuli for eight seconds and examined participants’ emotional responses during this time-frame. We were interested in the very first few seconds of viewing since, as highlighted by other authors (see e.g., Codispoti et al. 2001; Bradley et al. 2001), physiological and expressive reactions markedly occur within the first 6 s of viewing of affectively loaded pictures. From our experience (Dan-Glauser and Gross 2011, 2013, 2015; Thuillard and Dan-Glauser 2017), extending the original time frame from 6 to 8 s is a nice compromise to spot slower reactions like that of skin conductance or respiration, while permitting to record a significant number of trials for each condition. All pictures were shown with the same duration, size, distance and lighting.

### Measures

In order to have a comprehensive overview of the emotion reactions, we measured emotion responses in the three major emotion response domains: experience, expressivity, and physiological arousal (Kring and Gordon 1998; Matsumoto et al. 2007; Mauss et al. 2005).

#### Emotion Experience

Participants used a rating slider to continuously report their emotion experience (Variable Assessment Transducer, Biopac Systems, Inc., Goleta, CA, USA) throughout the picture presentation. The slider was unipolar with negative reports on the left side and positive reports on the right side. Output voltage (0–9 V) was extracted and converted into a negative and a positive scale (see the Data Extraction section for further details).

#### Emotion-Expressive Behaviour

Expressivity was assessed using bipolar surface electromyography (EMG). Electrodes were standard 4 mm Ag–AgCl sensors. Three EMG sites were recorded. Because of its reliable link with negative expressivity, left *Corrugator Supercilii* (Lang et al. 1993; Larsen et al. 2003) was the first targeted site. Left *Zygomaticus Major,* and left *Orbicularis Oculi* were the two chosen sites for positive expressivity. Zygomatic region is generally used for measuring positive expressivity, which motivated our choice to record this site. However, Zygomatic is not a completely direct measure of positive expressivity (Larsen et al. 2003). We thus decided to add a supplementary channel and target a region that is a reliable readout of Duchenne’s smile (Frank et al. 1993): *Orbicularis Oculi*. Electrode placement followed recommendations by Fridlund and Cacioppo (1986). Skin was first gently rubbed with NuPrep® gel (Weaver and Cie). Excess gel was removed with alcohol pads (Kendall Webcol® skin cleansing alcohol pads, Tyco healthcare). Finally, electrodes were filled with Signagel® (Parker Laboratories, Inc).

#### Autonomic and Somatic Responses

In order to examine different systems involved in autonomic reactivity, we measured cardiovascular, exocrine, and respiratory activities.

1. Electrocardiography (ECG): Three standard disposable pre-gelled Ag/AgCl electrodes were used for ECG recordings. One was placed approximately 5 cm below the lower rib on the left side of the abdomen. A second electrode was placed just below the right clavicle, along the mid-clavicular line. A third electrode, which functioned as a ground, was placed at the level of the C7 cervical vertebrae.
2. Electrodermal activity: Skin conductance level was recorded with two pre-gelled disposable Ag/AgCl sensors. They were placed on the thenar and hypothenar eminences of the non-dominant hand palm.
3. Respiration: Thoracic and abdominal respiration recordings were gathered with two respiration belts. The abdominal belt was placed around the waist, whereas the thoracic belt was placed high on the chest.

All parameters were recorded and amplified with MP150 compatible modules from Biopac Systems (Goleta, CA, USA). All sensors were from the same company. All acquired channels were sampled at 1000 Hz.

### Procedure and Design

Participants were tested in two sessions: first a questionnaire session, and then the main testing phase including the emotion regulation task. All participants gave informed consent to participate in the study. The procedure was reviewed and authorized by our regional ethical committee (CER-VD, protocol 2015–00,071), in accordance with the current national legal requirements (Ordinance on Human Research) and the latest version of the Declaration of Helsinki.

#### Session 1: Questionnaires

In the first session, participants came into the lab and completed the SF12 and the Edinburgh Handedness Inventory (see *Participant* section). They also completed other emotion-related questionnaires that served as pilot data for another project (unpublished data). After checking right-handedness and absence of health-related issues, participants were invited to register for the second session.

#### Session 2: Choice task

Participants returned for the emotion regulation task about 10 days after the first session. They were first informed about the procedure of the experiment and prepared for the physiological recordings. All instructions were presented on screen. They were told that we were interested in people’s reactions to different scenes and that they would see different emotional pictures. The rating dial was then introduced and we explained that the main task of the study was to report their feelings by moving the cursor while viewing each picture. A few training trials were used to familiarize participants with the rating system. They were then instructed about the emotion regulation task. The instructions were as follow: “Sometimes in this session (in some blocks), you will have the opportunity to choose yourself, among two options, what image you wish to see. Using the arrows, select the image topics, then return to the slider and concentrate on your feelings to report them.”. Participants again performed a few training trials. Since some trials were illusory choices, we were concerned that participants may believe that the program was malfunctioning and stop the experiment. To avoid this, we informed the participants that sometimes it was not possible for us to give them their choice.

All pictures were presented in a fully randomized order to control for habituation. All participants saw 10 blocks of pictures, four under the *Imposed* and six under the *Choice* conditions. Blocks were presented in a semi-randomized order with no more than two consecutive blocks of the same condition. At first, we had set the same number of trials in the *Imposed* blocks and *Chosen* blocks but while testing our experiment we realized that since choosing a picture takes more time than when a picture is imposed, the *Chosen* blocks were much longer than the *Imposed* blocks. Since we did not want differences between our conditions to be attributed to fatigue or boredom in longer blocks, we chose to reduce as much as possible the duration differences between block types. To do this, we attributed a different number of trials to different blocks, while maintaining an equal number of picture categories within each block. There were thus 24 trials in the *Imposed* condition blocks and 16 in the *Choice* condition blocks. The 24 trials of the *Imposed* blocks consisted of 12 positive and 12 negative pictures, each with three images of each content category. *Choice* condition blocks were composed of eight positive and eight negative pictures, each with two images of each content category. Of these 16 trials, six were non-respected choices (*Illusory Choice*) and ten were respected choices (*Situation selection*). The last *Choice* block differed in the number of trials. This was due to the pairing procedure: the program exiting the last *Choice* condition block once no more option for pairing was available (e.g., when all remaining pictures were of the same category).

**Fig. 2:**
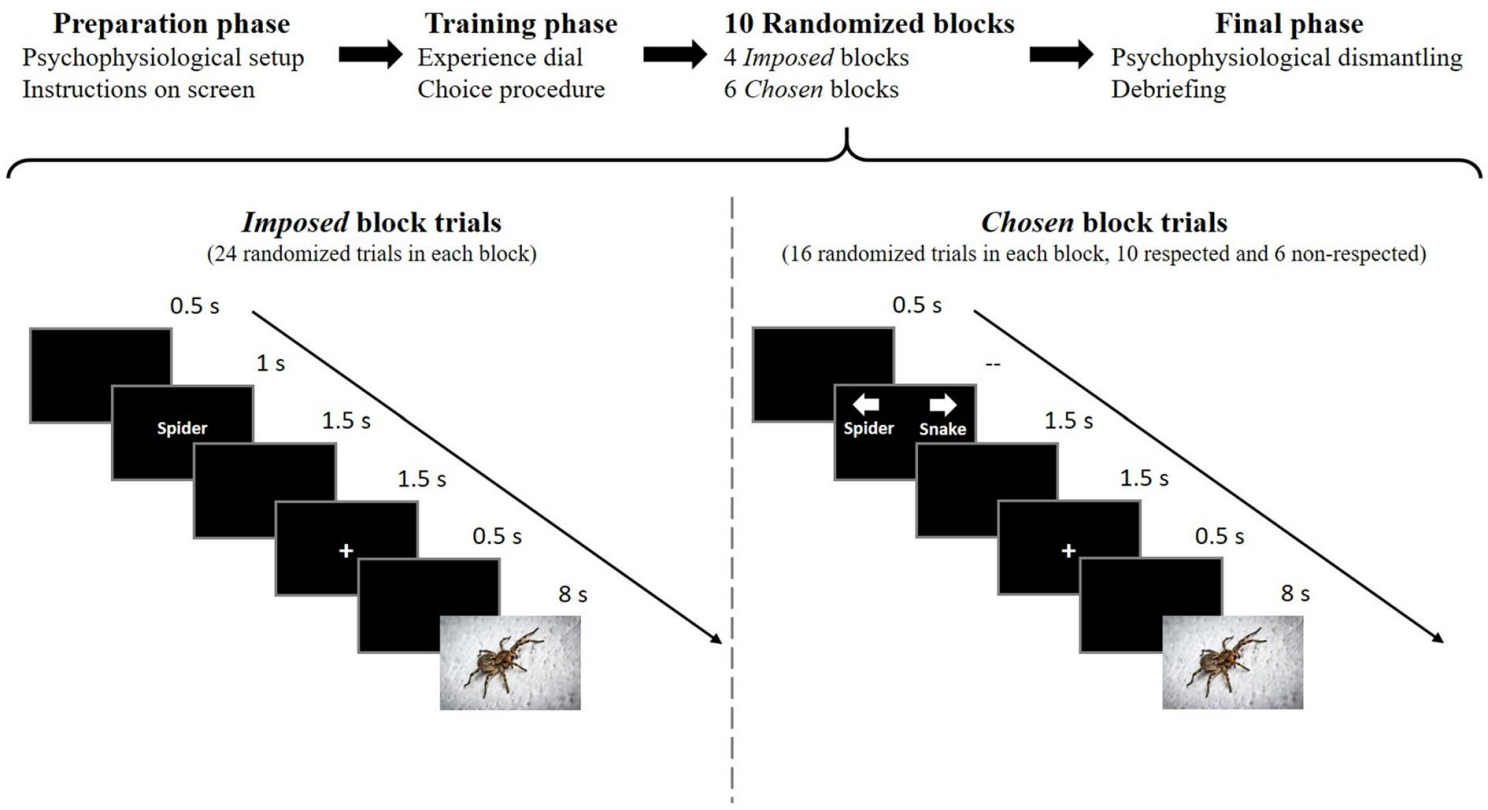
Illustration of the Session 2 procedure. An example of trial in each of the condition (*Imposed *or *Chosen*) is presented with the corresponding display duration of each screen

Each trial consisted of a blank screen (0.5 s), the choice screen, a blank screen again (1.5 s), a fixation cross (1.5 s), a blank screen again (0.5 s) and the picture presentation (8 s). In the *Imposed* condition, the choice screen was replaced by the display of the category of picture about to be presented (for 1 s). Only the images of the *Imposed* condition that were also seen in the *Choice* condition were retained for analysis in order to have a perfect match of intrinsic emotional and perceptive content in both conditions (see Fig. 1). After the computer session, sensors were removed and the participants were fully debriefed. The full procedure for Session 2 is depicted in Fig. 2.

### Data Reduction

All data were processed with Acknowledge 4.4 (Biopac System, Goleta, CA, USA). Some channels were bandpass filtered to increase the signal-to-noise ratio (20–500 Hz for EMG, 0.5–35 Hz for ECG, and 0.05–1 Hz for respiration). The channels were then manually scanned for movements or electric interferences, which were corrected by signal interpolation. To assess the temporal dynamics of emotional responses, the continuous parameters were segmented into 16 epochs of 0.5 s each. This segmentation was performed after the parameter extraction on the total duration of the recordings, which prevent signal distortion at the epoch boundaries. In addition to the 8 s picture presentation period, a baseline of 3.5 s was calculated for each trial and each parameter, spanning from 3.5 s before the picture presentation to the time of the picture onset. Thus, each trial had a baseline period that was used to normalize the response for some parameters (see below).

#### Emotional Experience

Ratings were exported to obtain mean values for each epoch. The recorded baseline served as the 0 point to calculate emotion intensity. Any value below the baseline was considered as a negative feeling and any value above as a positive feeling. Values were extracted as percentages, representing how far the slider was pushed between its 0 point (baseline) and its extreme values on either side. The data for each of the valence sides go from 0 = absence of emotional feeling to 100 = extreme emotion intensity.

#### Emotion-Expressive Behaviour

The EMG signal was rectified and smoothed (5 Hz) before being averaged for each epoch. Given the high variability in the contraction capacity of each individual, EMG values were expressed as the percentage of contraction with respect to the corresponding trial baseline (voltage recorded for a given time-frame / voltage recorded during baseline * 100) (Van Boxtel 2010; de Wied et al. 2006). Negative expressivity was measured with the *Corrugator* site. To parallel this negative expressivity with a single measure of positive expressivity, and given the high correlation found between the *Zygomaticus* and *Orbicularis* sites (across condition r = 0.35–0.45, *p* < 0.004), positive expressivity was measured with an average of the *Zygomaticus* and *Orbicularis* signal.

#### Autonomic and Somatic Responses

Heart rate was calculated from the ECG channel by transforming the inter-beat interval (duration between successive R waves). The skin conductance level was exported as mean values for each epoch. Respiratory rate and amplitude were calculated for each epoch. Respiratory rate was obtained by converting the duration of the cycle intervals into a number of cycles per minute (c/min). Respiratory amplitude was the difference in volts between the point of maximum inspiration and the point of maximum expiration. Given the high correlations between thoracic and abdominal respiratory rates (across condition *r* = 0.32–0.70, *p* < 0.009), these parameters were averaged. Similarly, thoracic and abdominal respiratory amplitudes were averaged for analyses. All these response channel data were calculated as the change in activity with respect to each trial baseline.

### Data Analyses

For each valence, trials were divided into four categories (see Fig. 1). In order to explore the dynamics of emotional responses, we also included time as a factor. Three runs of analyses were performed:

- We computed the descriptive values for each parameter under the *Imposed* condition, as a manipulation check for emotional induction.
- In order to replicate previous findings about *Situation selection* effect (Thuillard and Dan-Glauser 2017), we first focused on *Situation selection* only. ANOVAs were used with two within-factors: Regulation (comparing the *Imposed* condition, but only the matched trials *Imposed-respected*, and the *Situation selection* condition) and Time (16 epochs). Separate ANOVAs were performed for negative and positive trials for the following two reasons. First, contrasting positive and negative trials was not part of our research question. Second, previous research has shown different emotion (Lang et al. 1997; Palomba et al. 1997; VanOyen Witliet and Vrana 1995; Kensinger and Schacter 2006; Dolcos et al. 2004; Winton et al. 1984) and emotion regulation patterns (Hubert and de Jong-Meyer 1991; Mak et al. 2009; Kim and Hamann 2007) for positive and negative responses. Two effects were of interest in these analyses: (i) the main effect of Regulation, to assess the overall effect of choice, and (ii) the interaction effect Regulation x Time, particularly interesting to evaluate the *temporal dynamics* of choice effects.
- To investigate how the type of choice (*Illusory choice* or *Situation selection*) differentially impacted emotion responses, we conducted repeated-measure ANOVAs on each valence and parameter, investigating the effect of Regulation (*Imposed* vs. *Choice* conditions), the effect of Choice outcome (*Illusory choice* or regular *Situation selection*), and the effect of Time (16 time-frames). We were not interested in the reaction differences between avoided or chosen pictures (main effect of Choice outcome). Indeed, when given a choice, we already know that people tend to avoid negative pictures and choose pictures that are more positive. We did not focus either on Time main effect or on Time × Choice outcome interaction. Four effects were therefore of interest here: Regulation main effect, Time × Regulation interaction, Regulation × Choice outcome interaction (with simple effects focused on the regulation rather than on the choice contrasts), as well as the three-way interaction Regulation × Choice outcome × Time.

Greenhouse–Geisser corrections were applied where the assumption of sphericity was violated, and corrected degrees of freedom were reported in these cases. Effect sizes were reported using partial eta squares (η_p_^2^) and confidence intervals were reported where appropriate. *P*-values for interaction effects were all corrected for multiple comparisons with the Holm-Bonferroni criterion. Threshold for significance for all effects was set to 0.05 (two-tailed).

## Results

Table 1 shows the average reaction to the presented pictures in the *Imposed* condition for each calculated parameter.

**Table 1:** Sample size (N), Mean, SEM, and 95% Confidence Interval (CI) of experiential, expressive, and autonomic responses to negative and positive stimulations for all the imposed images

		Negative viewing			Positive viewing		
	N	Mean	SEM	Cl (95%)	Mean	SEM	Cl (95%)
Experience (/100)	64	44.61	2.61	[39.49; 49.74]	42.92	1.91	[39.17; 46.68]
Expressivity (EMG, %baseline)	64/62	137.07	6.00	[125.31; 148.84]	154.32	8.87	[136.93; 171.71]
Heart rate (∆bpm)	64	− 1.48	0.17	[− 1.80; − 1.15]	− 0.86	0.16	[− 1.17; − 0.56]
Skin conductance level (∆μS)	50	− 0.003	0.018	[− 0.04; 0.03]	− 0.05	0.01	[− 0.07; − 0.03]
Respiratory rate (∆c/min)	59	0.03	0.05	[− 0.06; 0.13]	0.11	0.05	[0.01; 0.20]
Respiratory amplitude ∆mv	64	− 0.006	0.017	[− 0.023; 0.011]	− 0.03	0.011	[− 0.052; 0.009]

The experience scale ranges from 0 (*no emotion*) to + 100 (*very negative/positive*). Expressivity is the percentage of baseline level, on N = 64 for negative viewing and N = 62 for positive viewing. All other parameters are expressed as differences with baseline level

We conducted two *t*-tests (one for the negative trials and one for the positive trials) that compared the experience mean to a 0-centered distribution. Both tests yielded significant results, *t*_(63)_ = 22.43, *p* < 0.001 for negative and *t*_(63)_ = 17.06, *p* < 0.001 for positive, confirming a successful emotional induction in the expected direction.

### The impact of Situation Selection

#### Experience

**Fig. 3:**
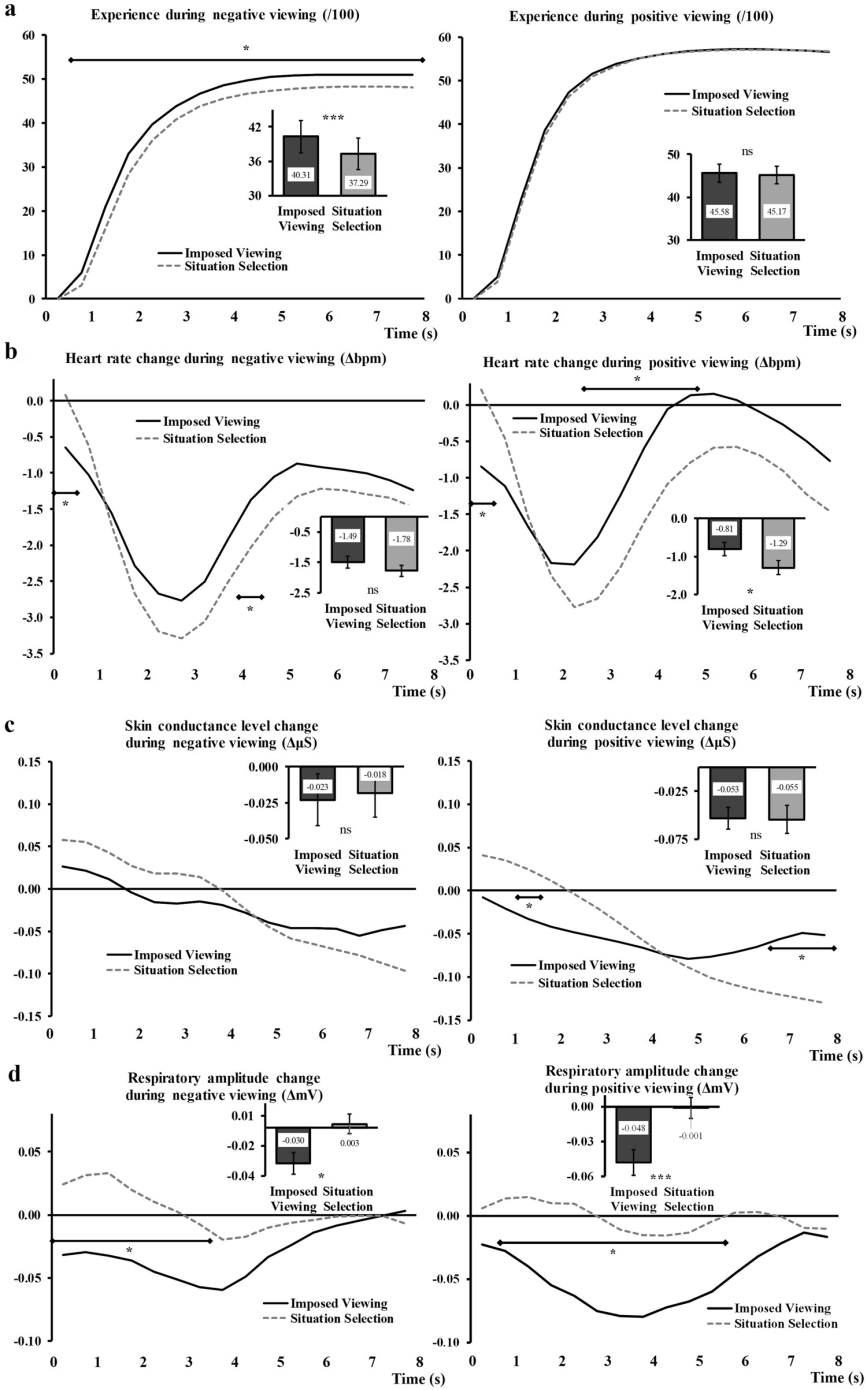
Impact of *Situation selection* on experience (**a**), heart rate (**b**), skin conductance level (**c**) and respiratory amplitude (**d**). For each parameter, the negative trials are shown on the left and the positive trials on the right. *Imposed* condition is represented with black continuous lines and *Situation selection* condition with grey dashed lines. The *Imposed* condition encompasses the responses to the exact same stimuli as the ones in the *Situation selection* condition (undistinguishable perceptual features and emotional content in these matched stimuli). Main effects are represented embedded, error bars are SEM. Significant contrasts are given with diamond-ended lines along the time course. ns = non-significant, *p < .05 with Holm-Bonferroni corrections, ***p < .001

During negative picture viewing, we observed a significant effect of Regulation, *F*_(1,63)_ = 21.92, *p* < 0.001, η_p_^2^ = 0.26, and a Regulation x Time interaction, *F*_(3, 178)_ = 3.97, *p* = *0.01*, η_p_^2^ = 0.06. When participants chose the image they wanted to see, this image triggered a negative experience (37.29) that was significantly lower than the reaction to the same image viewed without being chosen (40.31, see bar graph inserted in Fig. 3). The line graph in Fig. 3a (left side) shows the temporal unfolding of negative experience emergence over the 8 s of the picture presentation.

During positive picture viewing, the effect of Regulation was not significant, *F*_(1,63)_ = 0.62, *p* = 0.44. We also found no interaction Regulation x Time, *F*_(3, 181)_ = 1.47, *p* = 0.23. For the sake of comparison, we have nevertheless included this measure in Fig. 3a (right side).

#### Expressivity

The analyses performed on the negative trials did not show a significant effect of Regulation, *F*_(1,63)_ = 0.49, *p* = 0.49, nor a significant Regulation × Time interaction, *F*_(5,327)_ = 0.59, *p* = 0.71.

The analyses on the positive trials gave similar results, with no main effect of Regulation, *F*_(1,61)_ = 1.43, *p* = 0.24, and no significant Regulation x Time interaction, *F*_(5,290)_ = 1.2, *p* = 0.31.

#### Heart rate

For the negative picture viewing, the main effect of Regulation was not significant, *F*_(1,63)_ = 1.88, *p* = 0.18 but the interaction Regulation x Time was significant, *F*_(4,266)_ = 4.82, *p* = 0.001, η_p_^2^ = 0.07 (see Fig. 3b, left side).

Results on the positive pictures viewing recordings showed a significant effect of Regulation, *F*_(1,63)_ = 5.14, *p* = 0.027, η_p_^2^ = 0.08, and a Regulation x Time interaction, *F*_(3,196)_ = 9.60, *p* < 0.001, η_p_^2^ = 0.13. When participants chose the image they wanted to see, this image triggered a decrease in heart rate (− 1.29 bpm) that was significantly stronger than that recorded during imposed pictures viewing (− 0.81 bpm) (see Fig. 3b, right side).

#### Skin Conductance Level

The analyses performed on the negative trials did not show a significant effect of Regulation, *F*_(1,49)_ = 0.04, *p* = 0.85, but showed a significant Regulation × Time interaction, *F*_(3,140)_ = 6.73, *p* < 0.001, η_p_^2^ = 0.12 (see Fig. 3c, left side).

The analyses on the positive trials gave similar results, with no main effect of Regulation, *F*_(1,49)_ = 0.005, *p* = 0.94, but a significant Regulation × Time interaction, *F*_(2,86)_ = 14.84, *p* < 0.001, η_p_^2^ = 0.23 (see Fig. 3c, right side).

Bar graphs representing main effects (or absence thereof) have been inserted in Fig. 3c, for comparison with other parameters in the same conditions.

#### Respiratory Rate

The analyses on the respiratory rate showed no effect of the Regulation condition nor an interaction effect Regulation x Time. This was the case for negative viewing (main effect: *F*_(1,58)_ = 0.06, *p* = 0.80, interaction: *F*_(3,163)_ = 1.81, *p* = 0.15), as well as for positive viewing (main effect: *F*_(1,58)_ = 0.01, *p* = 0.98, interaction: *F*_(3,161)_ = 0.43, *p* = 0.72).

#### Respiratory Amplitude

During negative picture viewing, we observed a significant effect of Regulation, *F*_(1,63)_ = 6.38, *p* = 0.014, η_p_^2^ = 0.09, and a Regulation × Time interaction, *F*_(2, 120)_ = 5.34, *p* < *0.001*, η_p_^2^ = 0.08. When participants chose the image they wanted to see, this image triggered a slight increase in the respiratory amplitude signal (0.003 mV) that was significantly different from the decrease noted for imposed pictures (− 0.03 mV, see bar graph inserted in Fig. 3d, left side). The line graph in Fig. 3d (left side) shows the time course of this measure under both conditions.

During positive picture viewing, we also observed a significant effect of Regulation, *F*_(1,63)_ = 12.98, *p* = 0.001, η_p_^2^ = 0.17, and a Regulation × Time interaction, *F*_(3, 199)_ = 5.46, *p* = *0.001*, η_p_^2^ = 0.08. When participants chose the image they wanted to see, this image triggered virtually no change in the respiratory amplitude signal (− 0.001 mV) and this was significantly different from the decrease seen during imposed pictures viewing (− 0.048, see bar graph inserted in Fig. 3d, right side). The line graph in Fig. 3d (right side) shows the time course of this measure under both conditions.

### *Illusory Choice* Impact on Emotion Responses

#### Experience

**Fig. 4:**
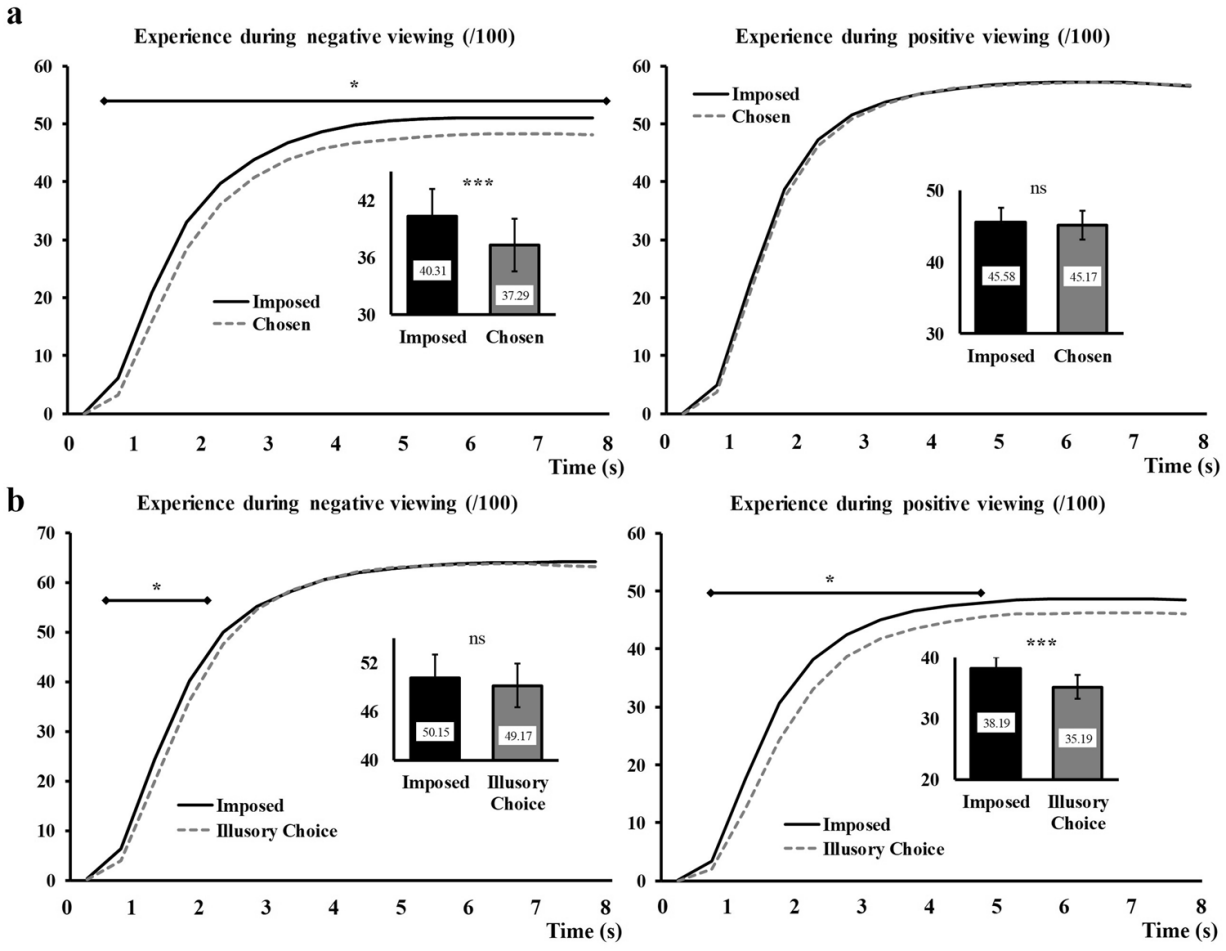
Illustration of the three-way interaction Regulation × Choice outcome × Time for emotional experience. Panel A illustrates the Regulation × Time interaction for the *Situation selection* condition. Panel B illustrates the Regulation × Time interaction for the *Illusory choice* condition. Negative viewing is shown on the left and positive viewing on the right. The *Imposed* condition is represented with black continuous lines and the *Situation selection* (**a**) or *Illusory Choice* (**b**) conditions with grey dashed lines. Main effects are represented embedded, error bars are SEM. Significant contrasts (Holm-Bonferroni corrected) are given with diamond-ended lines along the time courses. *ns* non-significant, *p < .05, ***p < .001

For negative picture viewing, we observed a significant main effect of Regulation, *F*_(1,63)_ = 18.43, *p* < 0.001, η_p_^2^ = 0.23. The targeted two-way interactions were both significant (Time × Regulation: *F*_(3,183)_ = 18.43, *p* < 0.001, η_p_^2^ = 0.10, Regulation × Choice outcome: *F*_(1,63)_ = 5.19, *p* = 0.03, η_p_^2^ = 0.08). Finally, the three-way interaction Time × Regulation × Choice outcome was also significant, *F*_(3,212)_ = 3.03, *p* = 0.03, η_p_^2^ = 0.05. Interpreting these results requires focusing on the three-way interaction. In order to address the impact of *Illusory choice*, the most meaningful way to conduct this analysis is to look for the Time x Regulation interaction at the two different levels of the Choice outcome factor, taking into account the mean square of the residual for the full model. The resulting statistics for these interaction effects were *F*_(3, 212)_ = 5.24, *p* = 0.002, η_p_^2^ = 0.07 for the *Situation selection* condition (respected choice) and *F*_(3, 212)_ = 11.2, *p* < 0.001, η_p_^2^ = 0.14 for the *Illusory choice* condition (non-respected choice). Figure 4a, b (left side) present these interactions. Collectively, our data suggest that negative experience is differentially impacted by *Situation selection* versus *Illusory choice* (compare Fig. 4a, b, left side). Indeed, in *Situation selection*, we recorded an emotional experience that was less negative than when the image was imposed, and this, during nearly all the visioning of the pictures. In contrast, when the choice was not respected (*Illusory choice*), the image still triggered a less negative experience but the reduction was only transient, occurring only within the first two seconds of the presentation (from 0.5 s to 2 s after the image onset).

For positive picture viewing, the very same significant effects as for negative viewing were observed: a Regulation main effect, *F*_(1,63)_ = 14.48, *p* < 0.001, η_p_^2^ = 0.19, a Time × Regulation interaction, *F*_(3,173)_ = 5.55, *p* = 0.002, η_p_^2^ = 0.08, a Regulation × Choice outcome interaction, *F*_(1,63)_ = 9.52, *p* = 0.003, η_p_^2^ = 0.13, and a three-way interaction Time × Regulation × Choice outcome, *F*_(3,184)_ = 3.55, *p* = 0.017, η_p_^2^ = 0.05. Like for negative pictures, we explored the three-way interaction by examining the Time × Regulation interaction at the two different levels of the Choice outcome factor, with full model residual correction. For *Situation selection,* the interaction effect was not significant, *F*_(3,184)_ = 1.35, *p* = 0.26. For *Illusory choice,* the interaction effect was significant, *F*_(3,184)_ = 11.53, *p* < 0.001, η_p_^2^ = 0.16. Figure 4a and b (right side) present these interactions. Collectively, these results suggest that *Situation selection* had no impact on the positive experience felt during positive situations (since chosen images triggered the same positive experience as when the same image was imposed, Fig. 4a right). On the contrary, *Illusory choice* made that image less positive (at least the first 5 s of viewing) than when the exact same image was seen in the *Imposed* condition (Fig. 4b, right).

#### Expressivity

Expressivity was not significantly affected by the tested factors. This was the case for negative viewing: *F*_(1,63)_ = 1.03, *p* = 0.32 (main effect of Regulation), *F*_(3,217)_ = 0.28, *p* = 0.87 (Time × Regulation), *F*_(1,63)_ = 0.05, *p* = 0.83 (Regulation × Choice outcome), and *F*_(4,257)_ = 0.67, *p* = 0.62 (three-way interaction Regulation × Choice outcome × Time); and for positive viewing: *F*_(1,61)_ = 1.39, *p* = 0.24 (main effect of Regulation), *F*_(4,258)_ = 0.96, *p* = 0.43 (Time × Regulation), *F*_(1,61)_ = 0.11, *p* = 0.74 (Regulation × Choice outcome), and *F*_(4,237)_ = 0.67, *p* = 0.61 (three-way interaction Regulation x Choice outcome × Time).

#### Heart rate

**Fig. 5:**
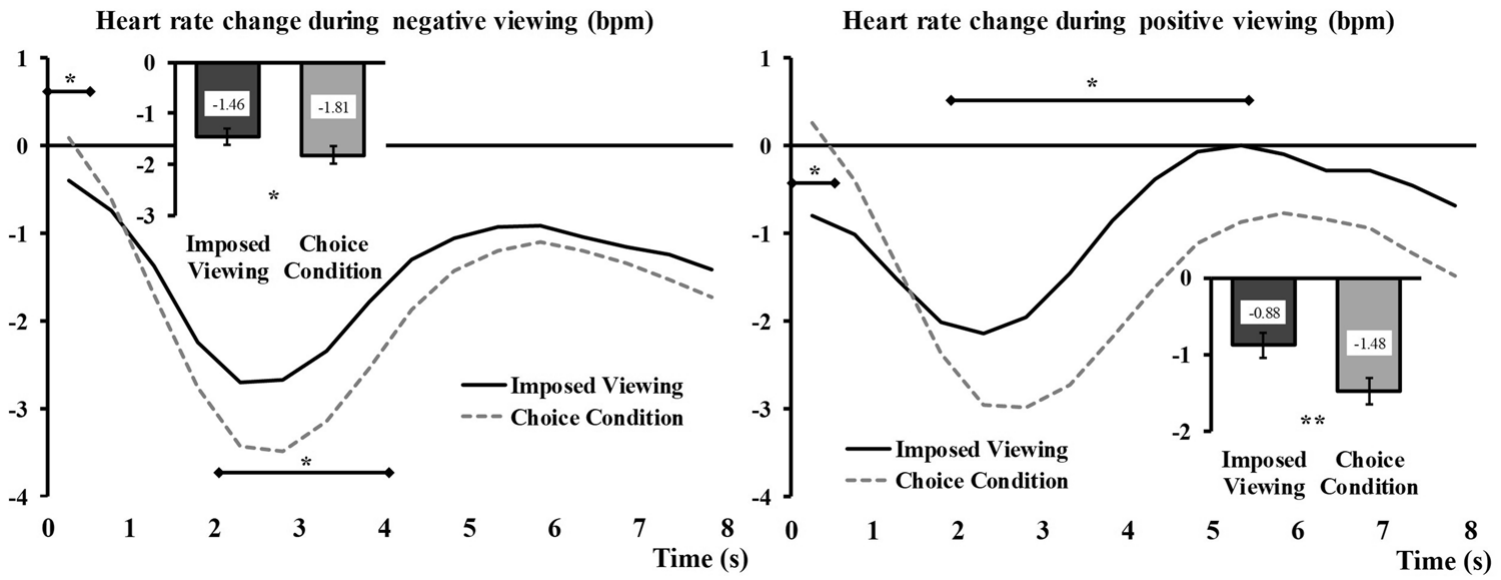
Illustration of the Time × Regulation interaction regarding changes in heart rate for negative viewing (left) and positive viewing (right). The levels of Choice outcome (*Illusory choice* or *Situation selection*) are aggregated in the *Choice* conditions (grey dashed lines) and contrasted together with the *Imposed* conditions (black continuous lines). Main Regulation effects are represented embedded, error bars are SEM. Significant contrasts (Holm-Bonferroni corrected) are given with diamond-ended lines along the time courses. *ns* non-significant, *p < .05, **p < .01, ***p < .001

For negative trials, analyses showed a significant main effect of Regulation, *F*_(1, 63)_ = 4.45, *p* = 0.04, η_p_^2^ = 0.06, and a significant Time × Regulation interaction, *F*_(4,221)_ = 5.86, *p* < 0.001, η_p_^2^ = 0.09 (see Fig. 5, left side). The Regulation x Choice outcome interaction and the three-way interaction were not significant, *F*_(1,63)_ = 0.27, *p* = 0.60 and *F*_(3,206)_ = 1.47, *p* = 0.22, respectively.

The same pattern of results was found for positive trials with a significant main effect of Regulation, *F*_(1, 63)_ = 8.73, *p* = 0.004, η_p_^2^ = 0.12, and a significant interaction Time × Regulation, *F*_(3,219)_ = 18.76, *p* < 0.001, η_p_^2^ = 0.23 (see Fig. 5, right side). The Regulation × Choice outcome interaction and the three-way interaction were not significant, *F*_(1,63)_ = 0.74, *p* = 0.39 and *F*_(4,242)_ = 0.81, *p* = 0.51, respectively.

Collectively, these results suggest that heart rate responses shared commonalities, whether the choice was respected or illusory. Globally, we see that, when people could choose the situation, their orienting response to the stimulation was subsequently stronger (see values of the main effects: − 1.81 bpm for negative and − 1.48 bpm for positive stimuli) than when this task was not given (see values of the main effects: − 1.46 bpm for negative and − 0.88 bpm for positive stimuli). Importantly, this was true regardless of whether choice was respected or not. Detailed dissection of the response time course with Holm-Bonferroni corrected contrasts (Fig. 5) showed that this was true particularly for the first few seconds after the image onset, and did not persist until the end of the viewing, which speaks in favour of a modulation of the orienting response.

#### Skin conductance Level

**Fig. 6:**
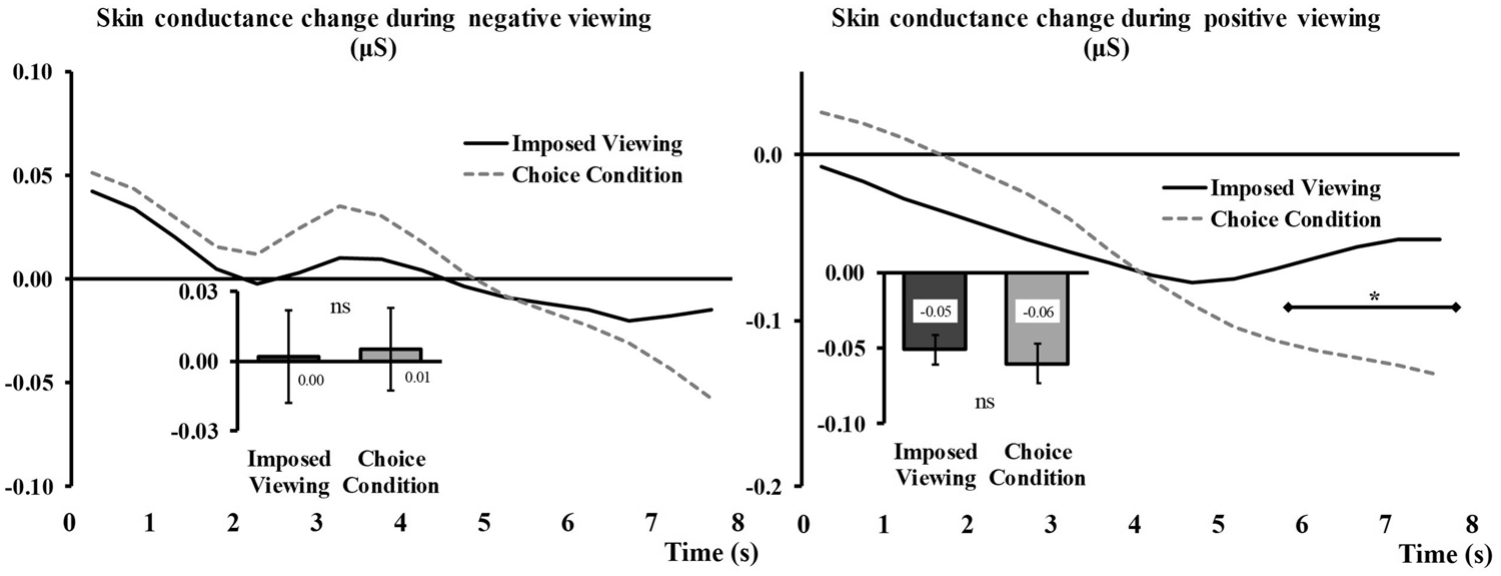
Time × Regulation interaction for skin conductance changes during negative viewing (left, no significant effects) and during positive viewing (right). The levels of Choice outcome (*Illusory choice* and *Situation selection* conditions) are aggregated to form the *Choice* conditions (grey dashed lines) and contrasted to all the *Imposed* trials (black continuous lines). Main Regulation effects are represented embedded, error bars are SEM. Significant contrasts (Holm-Bonferroni corrected) are given with diamond-ended lines along the time courses. *ns* non-significant, *p < .05

None of the analyses conducted on the negative trials gave significant results, *F*_(1,49)_ = 0.05, *p* = 0.83 (main effect of Regulation), *F*_(2,76)_ = 1.50, *p* = 0.23 (Time x Regulation), *F*_(1,49)_ = 0.00, *p* = 0.95 (Regulation × Choice outcome), and *F*_(1,73)_ = 2.14, *p* = 0.14 (three-way interaction). For the sake of comparison with Fig. 3, the data related to this testing are presented in Fig. 6, left side.

For positive trials, we found a significant Time x Regulation interaction, *F*_(2,86)_ = 14.56, *p* < 0.001, η_p_^2^ = 0.23. This interaction effect shows that, toward the end of the viewing period, the skin conductance measured in the Choice condition was lower than in the *Imposed* condition (Fig. 6, right). This effect occured no matter if the choice was respected or not (no triple interaction found, *F*_(2,112)_ = 0.89, *p* = 0.43). The other relevant effects were not significant, *F*_(1,49)_ = 0.41, *p* = 0.53 (main effect of Regulation), *F*_(1,49)_ = 0.42, *p* = 0.52 (Regulation x Choice outcome).

#### Respiratory Rate

**Fig. 7:**
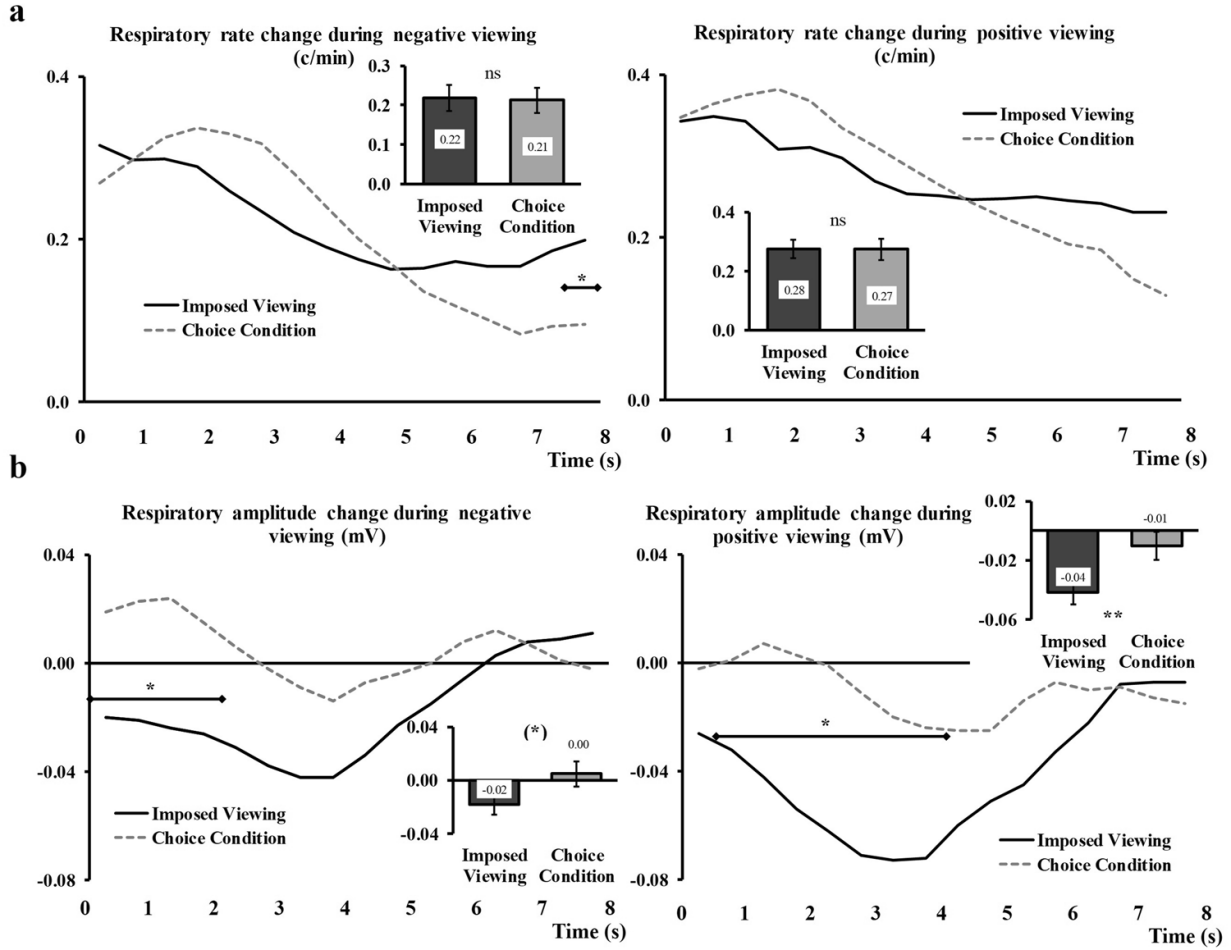
Illustration of Time × Regulation interactions for respiratory rate (**a**) and amplitude (**b**). For each parameter, the negative trials (left) and the positive trials (right) are depicted. The levels of Choice outcome (*Illusory choice* and *Situation selection* conditions) are aggregated to form the *Choice* conditions (grey dashed lines) and contrasted to all the *Imposed* trials (black continuous lines). Main effects are represented embedded, error bars are SEM. Significant contrasts are given with diamond-ended lines along the time course. *ns* non-significant, (*)*p* = .06, *p < .05, and **p < .01 with Holm-Bonferroni corrections

For negative trials, we found a significant Time x Regulation interaction, *F*_(3,162)_ = 4.47, *p* = 0.006, η_p_^2^ = 0.07. Contrast analyses for this effect (Fig. 7a, left side) indicate that a lower respiratory rate is reached at the end of the examined time windows for pictures viewed under the *Choice* conditions (regardless of whether the choice was respected), as compared to the same pictures viewed under the *Imposed* conditions. All other relevant effects were not significant, *F*_(1,58)_ = 0.02, *p* = 0.89 (main effect of Regulation), *F*_(1,58)_ = 0.04, *p* = 0.84 (Regulation × Choice outcome), and *F*_(3,148)_ = 0.81, *p* = 0.47 (three-way interaction).

For positive trials, the Time × Regulation interaction was only marginally significant *F*_(3,151)_ = 2.77, *p* = 0.05, η_p_^2^ = 0.05 (for illustration purposes, this interaction can be seen in Fig. 7a, right side). All other relevant effects were not significant, *F*_(1,58)_ = 0.00, *p* = 0.94 (main effect of Regulation), *F*_(1,58)_ = 0.01, *p* = 0.91 (Regulation × Choice outcome), and *F*_(3,154)_ = 1.58, *p* = 0.20 (three-way interaction).

#### Respiratory Amplitude

For negative trials, we found a significant Time × Regulation interaction, *F*_(3,162)_ = 4.47, *p* = 0.006, η_p_^2^ = 0.07. Contrast analyses for this effect (Fig. 7b, left side) suggest a lower respiratory amplitude reached during the first two seconds for pictures viewed under the *Imposed* condition, as compared to the exact same pictures viewed under the *Choice* conditions (regardless of whether the choice was respected or not). The main effect of Regulation was only marginally significant, *F*_(1,63)_ = 3.73, *p* = 0.06, η_p_^2^ = 0.06. All other relevant effects were not significant, *F*_(1,63)_ = 1.25, *p* = 0.27 (Regulation x Choice outcome), and *F*_(2,153)_ = 0.89, *p* = 0.43 (three-way interaction).

Analyses on positive trials showed a significant main effect of Regulation, *F*_(1, 63)_ = 6.53, *p* = 0.01, η_p_^2^ = 0.09, and a significant interaction Time × Regulation, *F*_(3,218)_ = 8.59, *p* < 0.001, η_p_^2^ = 0.12. Contrast analyses for this latter interaction (Fig. 7b, right side) suggest a lower respiratory amplitude reached during the first four seconds for pictures viewed under the *Imposed* condition, as compared to the exact same pictures viewed under the *Choice* conditions (regardless of whether the choice was respected or not). The Regulation × Choice outcome interaction and the three-way interaction were not significant, *F*_(1,63)_ = 1.53, *p* = 0.22 and *F*_(3,195)_ = 1.14, *p* = 0.33, respectively.

## Discussion

With the present research, our aim was twofold. First, we wanted to replicate the results of a previous study (Thuillard and Dan-Glauser 2017). Second, we wanted to better understand what makes *Situation selection* an efficient emotion regulation strategy. Indeed, if we plan to promote the implementation of *Situation selection* in everyday situations for particular populations, we must gain a deeper understanding of how choice regulates emotions. More particularly, it is crucial to know whether choice has to be respected in order for it to be efficient. If this is the case, implementation of *Situation selection* should occur only if we can follow the proposed choice. On the contrary, if choice only has much more importance than the resulting situation, following up on the choice is not mandatory for *Situation selection* to successfully regulate emotions. To answer to this point, we have adapted a previous protocol to carefully examine *Choice* conditions in which we can distinguish between respected and non-respected choices. More importantly, and in order to eliminate differences between chosen and avoided options in terms of content, we designed an analysis procedure that allowed us to compare the responses to an identical picture in a *Choice* condition (whether respected or not) and in an *Imposed* condition. Finally, given the importance of response dynamics in emotion processes, we included time as a factor to be able to discuss even transient effects.

With respect to our first aim, and as expected, we found that *Situation selection* effects on emotional experience were consistent with previous findings (Thuillard and Dan-Glauser 2017). Indeed, we confirmed that choosing the upcoming emotional situation significantly decreased negative experience, but left positive emotional experience unaffected. Past work has emphasized that self-motivation and sense of autonomy are being greatly influenced by freedom of choice (Deci and Ryan 2000). Here, by analysing the short and immediate affective reactions in *Situation selection* condition, we can propose that a possible functioning mechanism of this strategy may lie in the freedom of choice that a participant has with respect to an upcoming emotional situation. This possibility is particularly supported by our results, since the differences in experience are noticed for the same situations (same images), the only difference between the conditions lying in the fact of having the choice or not.

Concerning expressivity, we replicated the absence of *Situation selection* impact for positive situation, but did not find the previously reported expressivity enhancement in negative viewing. The absence of positive correlation between experience and expressivity in both studies contradicts the supposed coupling and coherence between experiential and expressive emotional reactions (Buck 1980; McIntosh 1996). It is however consistent with previous literature arguing that emotion regulation may disrupt the coupling between channels (Dan-Glauser and Gross 2013; Hollenstein and Lanteigne 2014). Hence, different emotion regulation strategies may not uniformly impact all emotion responses, but may have preferred entry points. Hence, although experience and physiology are impacted by *Situation selection*, expressivity might not be, or at least not systematically. Another explanation may be that performing a choice represents a greater cognitive effort than when a situation is imposed. Potential reduction in expressivity may thus be compensated in the *Choice* condition by additional muscular contraction driven by mental effort (Boxtel and Jessurun 1993).

With respect to physiological arousal, we found results that are similar to the previous study. Indeed, our present results confirm a stronger orienting response in the *Situation selection* condition for the heart rate parameter (in the first few seconds after the emotional picture onset), coupled with a decrease in skin conductance level at the end of the recording period. Regarding respiration, we found a reduction in respiratory amplitude in the *Imposed* condition, whereas in the *Situation selection* condition participants showed a rather stable amplitude throughout the viewing. We thus confirm a more aroused respiratory pattern in the *Imposed* condition than in the *Situation selection* condition. Overall, it appears that the choice mechanism involved in *Situation selection* helps regulating emotions by decreasing negative experience and sympathetic excretive function, and slightly enhancing cardiovascular reactivity.

With respect to the second aim, there are two main results to discuss. First, and contrary to what we expected, three-way interactions between our factors of interest are present only in the experiential domain. Further analyses showed that when choice was not respected, *Situation selection* lost its regulatory function on negative emotional experience. Hence, if we focus on subjective emotional experience as felt by the participants, offering a choice that cannot be followed has deleterious consequences on the experiential outcome, cancelling the benefit induced by giving the choice. However, and contrary to what could have been further expected, a non-respected choice does not trigger additional negative feelings. Thus, on average, giving a choice will trigger less negative experience than imposing a negative situation. However, a non-respected choice in positive situations will trigger less positive experience than when it is imposed. Extrapolating this idea to a daily life example we can for instance argue that when we choose a meal in a restaurant that turns out to be unavailable, we would appreciate the replacement dish less than if this dish was the only available option in the first place.

A second important result regards the physiological parameters. We observed that none of the parameters showed a three-way Time × Regulation × Choice outcome interaction, which is congruent with our expectations. Instead, most of the results showed that stimuli seen in the *Choice* conditions triggered less physiological arousal than the *exact same* stimuli seen in an *Imposed* condition, regardless of whether the choice was respected or not. This is especially true for skin conductance and respiratory activity results. While we cannot equate absence of interaction with evidence of no effect, and that habituation effect may have blunted existing differences, the fact that both respected and non-respected choices converged to decrease arousal is in contradiction with our second proposed option that not getting what we choose could have triggered a strong increase in emotional arousal instead of the expected regulation-induced decrease. An absence of major emotional rebound effect (increased emotional arousal when choice is not respected) is in itself an important finding in the light of offering emotional choices in daily life. Having the choice may thus lower body reactivity, at least in the short term, and counteract emotional arousal in people facing emotional stimulations. At the time the present study was completed, a study by Vujovic and colleague (Vujović and Urry 2018) showed that people do not compensate with another emotion regulation strategy when *Situation selection* has failed. Interpreting their results in combination with ours, we propose that this absence of need to further regulate emotions is due to *Situation selection* (whether a success or not) being already regulatory on some emotional responses (e.g., physiological).

By mostly replicating the results that presented *Situation selection* as an efficient emotion regulation strategy, we confirm that some emotional responses may be down-regulated when choosing an emotional situation. However, this is not the case with heart functioning, which shows a stronger orienting effect in the *Choice* condition, regardless of whether choice is respected or not. This may indicate that *Choice* condition is sufficiently challenging to trigger an orienting response (Graham and Clifton 1966; Sokolov 1963; Lang et al. 1993). Alternatively, we could see here a manifestation of emotion regulation. Indeed, recent literature proposed that orienting responses may be linked to the choice of a particular strategy to implement (Ghafur et al. 2018). The choice to regulate emotions and the choice of an upcoming situation in order to regulate emotions may thus share common processes that are reflected in similar cardiovascular response patterns.

Several limitations can be highlighted regarding this study. First of all, choice is limited to two options, which is very particular as we cannot really disentangle whether the choice is made according to preference for the chosen option, or avoidance of the alternative. The choice given to participants would deserve to be extended to a less limited and more ecologically valid set of options. A second limitation is the unbalance between respected vs. non-respected trials in order to reduce expectations regarding the image that was going to be presented. This is associated with the more general limitation that we do not know how the participants’ expectations vary with these instructions. Indeed, they know in advance that sometimes there will be non-respected trials. The first impulse to address this limitation would be to think that between-subject design would be more appropriate, with one group having respected choice and the other non-respected choice. We believe that this is not the case. Indeed, in addition to the limitation of comparing different physiologies of different people, we would face the reaction of participants always placed in the non-respected condition. In such a setting, they would quickly turn to selecting the option they want to avoid in order to get their choice. Future studies could also consider creating designs combining within and between subject analyses in order to deal with these important intertwined concepts. In particular, between-subject testing of several combination of respected and non-respected trials would permit to eliminate the expectations regarding the upcoming trials and would permit to lessen the habituation necessarily induced by the high number of trials necessary in within-subject designs. Third, we considered here broad valence affects (positive and negative) as raised by dimensional theorists of affect (Posner et al. 2005; Barrett et al. 2007; Russell 1980). This distinction has the advantage of limiting the problem of boundaries between emotion categories, which are linked to the frequent occurrence of mixed emotions (Ellsworth and Scherer 2003). Still, we admit that this approach cannot inform on the efficiency of *Situation selection* for particular emotions such as joy, fear, anger or sadness. Further investigations on the type of affect induced may provide more guidance for implementing *Situation selection* in daily situations. Finally, we could notice in our results that differences between our conditions could occur at different points in time. For example, differences could be transient in the first part of the viewing and not be present at the end of the recorded window (for heart rate or respiratory amplitude, for example), while other differences seem to emerge rather late in the process (for skin conductance or respiratory rate, for example). For this latter case, it remains open whether these differences persist beyond these 8 s post-stimulation threshold. It will therefore be crucial in future studies to specifically address the significance of this dynamic differences and uncover the biological mechanism and relevance the switch (if any) between transient and prolonged effects of emotion regulation.

This study investigated whether the effect of *Situation selection* goes beyond the choice we make and occurs irrespectively of the success of the selection. We confirmed that the choice process is determinant in the regulation effect for some physiological measures of emotion such as skin conductance and respiration. However, we did not find a regulatory effect for negative emotional experience when *Situation selection* is not followed by the chosen situation. Nevertheless, even though *Illusory choice* loses the positive effect of choice and does not reduce negative experience, it does not increase it either. This point is essential when reflecting upon implementing a decision or an action in order to regulate emotion in daily life situations or for advising it in case of patient emotion regulation coaching. For this latter case, particular attention should be given to the concept of uncontrollability, which could be especially triggered by *Situation selection* not being followed by the chosen option, and could lead the patient to abandon this strategy altogether. Particular attention on this matter should be given to patients with depressive pathology and low psychological flexibility (Trindade et al. 2020). Altogether, our study nevertheless confirms that the resulting situation is not the only factor that matters to regulate emotion and that a much more complex mechanism drives *Situation selection* efficiency. In particular, what seems to be an important aspect is to feel in control of our emotional future…no matter if this is the case, or not.
